# Phyllodes Tumors of the Breast: A Literature Review

**DOI:** 10.7759/cureus.10288

**Published:** 2020-09-07

**Authors:** Musaed Rayzah

**Affiliations:** 1 Department of Surgery, College of Medicine, Majmaah University, Majmaah, SAU

**Keywords:** phyllodes tumors, breast, fibroadenoma, fibroepithelial neoplasms, management of phyllodes tumor, cystosarcoma phyllodes

## Abstract

Phyllodes tumors (PTs) of the breast are considered a rare fibroepithelial neoplasms of the breast and are considered a challenging for both pathologists and surgeons. The World Health Organization (WHO) has classified PTs histologically as benign, borderline, and malignant. PTs can be detected in all ages; however, the median age of presentation is 45 years. PTs can mimic fibroadenoma in clinical presentations. Breast imaging is also similar to fibroadenomas. Cytological diagnosis of PTs by biopsy is usually unreliable. However, a core needle biopsy is superior to fine-needle aspiration. Surgery is considered the mainstay treatment for PTs of the breast with a goal of achieving negative margins. Adjuvant chemotherapy and radiation therapy use for malignant PTs are controversial.

## Introduction and background

Phyllodes tumors (PTs) of the breast are an infrequent fibroepithelial neoplasm that accounts for less than 1% of all breast neoplasm. The incidence of PTs is low 0.3%-0.9% of all breast tumors [1,2]. PTs were initially described by Muller in 1838 as Cystosarcoma phyllodes. Phyllodes derive from the Latin Phyllodium which means ‘leaf-like’ based on a gross pathological description of a leafy, bulky, cystic, and fleshy tumor of the breast [3]. In 1982, the World Health Organization (WHO) has classified PTs histologically as benign, borderline, and malignant based on their histopathologic characteristics, which has been accepted widely (Table 1) [4]. Benign PTs are more frequent, constitute between 35% and 64%, borderline PTs between 7% and 40% of cases, while malignant PTs reach up to 30% [5,6]. Triple assessment by clinical, radiological, and histological examination are the initial assessment to evaluate PTs. It is difficult to differentiate PTs from other breast tumors before surgical excision. It has unpredictable behavior regardless of its histological grade. Local recurrences and distal metastasis rarely occur in benign PTs, while common in borderline and malignant PTs. Surgery with clear resection margins remains the mainstay treatment for PTs of the breast. Local recurrence is reported to be approximately 8% for benign PTs and 21% for borderline cases [7].

**Table 1:** Phyllodes tumor classifications subtypes HPF, high power field

	Benign	Borderline	Malignant
Stromal hypercellularity	Minimal	Moderate	Marked
Cellular pleomorphism	Minimal	Moderate	Marked
Mitosis	0–4/HPF	5–9/HPF	>10/HPF
Margin	Pushing	Pushing or infiltrating	Infiltrating
Stromal pattern	Uniform	Heterogeneous	Marked

## Review

Epidemiology and risk factors

PTs are considered rare tumors. Because of limited data, the etiology of PTs is unknown, and the risk factors are not yet clearly identified; however, Latin women and East Asians who were born in Central or South America and living in the United States have a higher risk [8-10]. Additionally, genetic mutations in the chromosomal regions of +1q, +5p, +7, +8, −9p, −10p, −6, and −13 correlated with borderline and malignant PT of the breast [11]. Few studies have exhibited the association between family relatives and PTs [12,13]. Women with Li-Fraumeni syndrome have an increased risk for PTs [14]. PTs occur almost exclusively in females; however, a few cases have been reported in men, all of which were associated with gynecomastia [15,16]. The median age of presentation in PTs is 45 years, with age ranging between 9 and 93 years [8,17], with Asians diagnosed at a significantly earlier age than other groups [18].

Clinical presentation and diagnosis

PTs usually present clinically as a benign breast mass, with rapid growth sometimes. In some patients, the lesion may present with rapid growth after being present for many years. It can associate with blue discoloration, dilated skin veins, skin ulcers, nipple retraction, and palpable axillary lymph nodes in rare cases [17-19]. It rarely involves the nipple-areola complex or causes ulceration to the skin. The most common presenting symptom is a breast lump, usually located at the upper outer quadrant of the breast, and rarely bilateral in 1.8% [15]. The size of PTs varies between 0.5 and 30 cm with a mean between 5 and 7.2 cm [20,21].

Triple assessment including, clinical, radiological, and histopathological evaluations of suspected breast lumps are considered to be the standard of care. By ultrasound, it appears as a solid mass, inhomogeneous, with a radiolucent halo, lobulated border, and sometimes coarse microcalcifications. The presence of a solid mass with multiple or single, round or cleft like cystic spaces with posterior acoustic enhancement suggest the diagnosis of PT, but Intramural cysts with the absence of posterior acoustic enhancement can also be present. High vascularity is usually present in solid components. On mammographic imaging, they emerge as hyperdense, large, round, or oval, well-circumscribed lesions [22,23]. There is no clear indicator of malignancy observed on either ultrasounds or mammography, most of the time they have features similar to fibroadenoma on mammography and ultrasonography, however, with a higher mammographic density for PTs [24]. Even though magnetic resonance imaging (MRI) is considered to be extremely sensitive for the detection of breast cancer, it is still difficult to differentiate PTs from other breast tumor types [25,26]. On MRI, they are seen as oval, round, or lobulated masses with circumscribed margins as with the mammography. Although it is still difficult to differentiate phyllodes tumors from other breast tumors, PTs have higher signal intensities on T1-weighted images and lower or equal signal intensity on T2-weighted images than normal breast parenchyma [17,27]. The role of MRI in diagnosing PTs still under argument and not yet understood, although some authors have found evidence suggesting that MRI may have a high concordance rate with histopathology [17,27].

Cytological diagnosis of PTs by biopsies is usually unreliable [17,28]. Diagnosis of PTs by fine-needle aspiration cytology (FNAC) is difficult [17,29], as it is unreliable in distinguishing PTs from fibroadenoma cytologically. Scolyer et al. [29], compared the cytology of PTs and fibroadenomas, and concluded that if hypercellular stromal fragments are seen on FNAC, the possibility of PT should be raised and excision recommended. Foxcroft et al. [28], reviewed 83 cases of PTs, found cytology proposed PT in only 23% by FNA guided biopsies, while it was 65% of PTs on core biopsy.

Pathology

PTs are fibroepithelial tumors characterized by epithelial and stromal proliferation. On gross examination, PT mimics the fibroadenoma, but in the cut surface exhibits cleft like spaces with distributed nodular stromal growth, and the color differs from tan to yellowish gray. Also, stromal overgrowth, mitotic activity, and increased stromal cellularity are present [30]. Hemorrhage and necrosis can be seen in malignant type, also, a malignant type can have a fleshy sarcoma-like cut surface which is softer than benign PTs or fibroadenoma. Histologically, PTs are classified into different grades by the World Health Organization to determine their prognosis and clinical behavior [4]. These include benign, borderline, and malignant PTs based on histologic criteria, which include stromal cellularity, degree of cellular pleomorphism, mitotic activity, tumor margin, and stromal pattern (Table 1) [4]. Benign PTs constitute between 35 % to 64 %, whereas the malignant form accounts for about 25 % of cases [5,6,31]. A benign PT is characterized by well-defined tumor borders, mild stromal cellularity, none to mild atypia, < 5 mitotic figures per 10 high power field (HPF), and lack of stromal overgrowth or malignant heterologous components [32]. A borderline PT is characterized by typically well-defined or focally permeative tumor borders, absent or focal stromal overgrowth, moderate stromal cellularity, mild or moderate stromal atypia, and no malignant heterologous components [32]. Mitotic activity is in the range of 5-9 per 10 HPF. A malignant PT characterized by marked stromal cellularity and atypia, permeative margins, stromal overgrowth, and mitotic activity of at least 10/10 HPFs [33].

Treatment

Surgery is the mainstay treatment for PTs of the breast. However, due to their unclear clinical presentation, vague pathological behavior, and difficult preoperative diagnosis, there still seems to be a predicament in their treatment plans. In the past, simple mastectomy was the recommended treatment for borderline and malignant PTs. Breast-conserving surgery (BCS) was safe and adequate even for malignant PTs if complete excisions achieved [34,35]. The extent of surgery remains arguable because the surgical resection margin is thought to be associated with the local recurrence of PTs. Also, numerous clinical studies recommend wide excision of the tumor with 1 cm clear margin [22,31,36], which can cause a major difficulty in achieving good cosmetic results. However, recent studies show that there is no direct relationship between local recurrence rate and the width of negative margins [21,37]. Jang et al. reviewed 164 PT cases, found that the only factor that strongly predicted local recurrence was the presence of tumor cells on the resection margin, while the width of the resection margin did not correlate with local recurrence risk [21]. Onkendi et al. have shown that disease-free survival was not affected by the extent of surgical resection in patients with borderline and malignant PTs [37]. For benign and borderline PTs have a less aggressive disease course and the recurrence rates are low regardless of the resection margin status [38,39].

**Figure 1:**
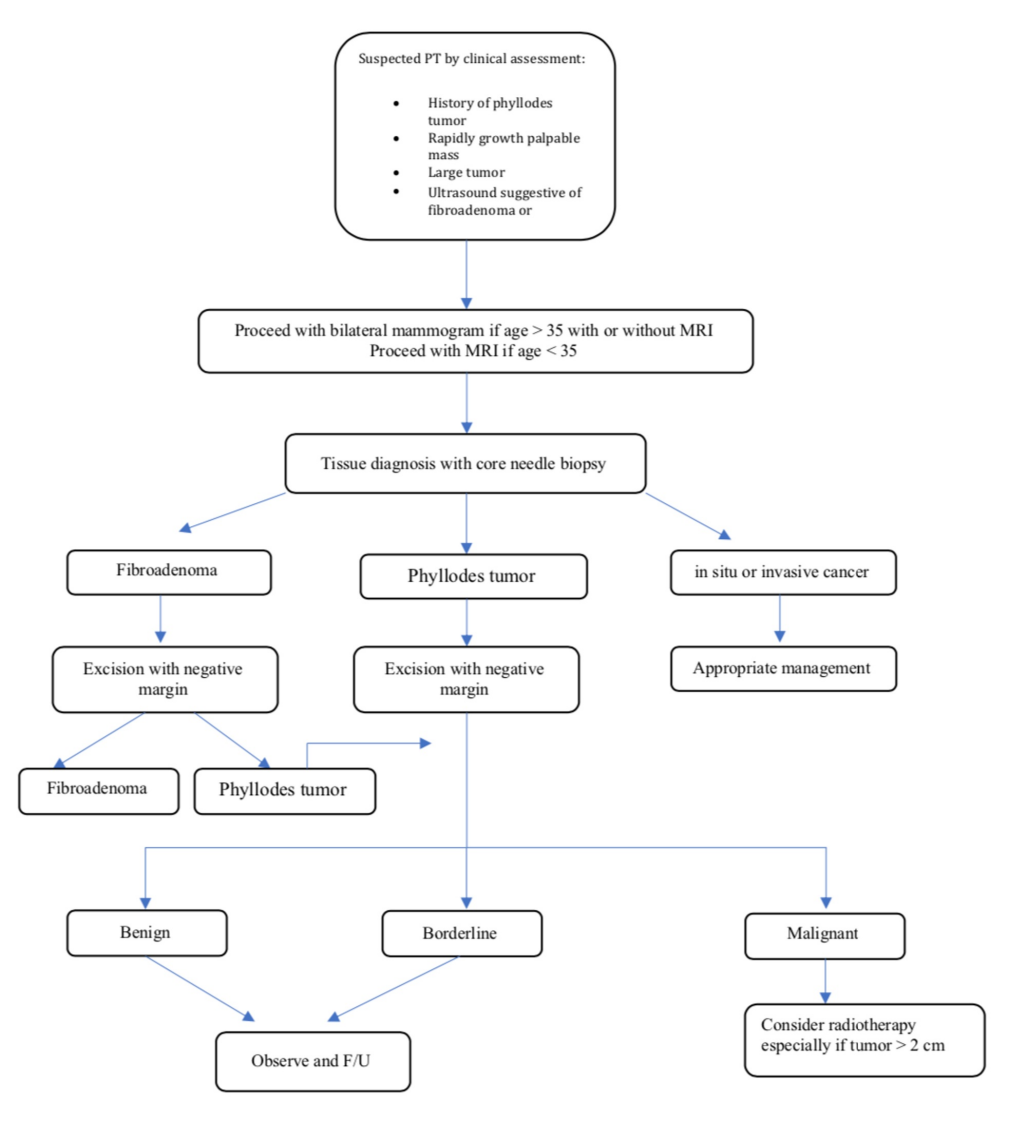
Management algorithm of PTs PTs, phyllodes tumors

Adjuvant radiation therapy use for malignant PTs is controversial [19,36,40]. Gnerlich et al. [41] in an analysis of cases collected from National Cancer Data Base from 1998 to 2009, demonstrated that there were an increase in time to LR and a significant decrease in LR in women who received adjuvant radiotherapy in comparison to those women who had surgery alone for malignant PTs but without a significant improvement in disease-free or overall survival. Belkacemi et al. [42] demonstrated that adjuvant radiotherapy for borderline and malignant PTs yielded a superior 10-year local control rate (86% with radiation versus 59% without radiation). Also, Barth et al. [7] found that no local recurrence was observed after a median follow-up of 56 months for women who received breast conservative surgery and adjuvant radiotherapy for borderline and malignant PTs with a confirmed margin-negative. However, the current National Comprehensive Cancer Network (NCCN) guidelines recommend consideration of radiotherapy for malignant phyllodes only in the setting of local recurrence (level 2B evidence) [43]. Adjuvant chemotherapy is more controversial and its effect in PTs is lacking. Adjuvant cytotoxic chemotherapy lacks the evidence of providing benefits in reducing local recurrences or improvement in disease-free or overall survival death. However, it can be considered for large tumors, when adjacent structures such as the chest wall are involved, or unresectable distant metastases [44]. Endocrine therapy is not proven to have an effect in PTs, although pathologically contain estrogen receptors in 58%, and progesterone receptors in 75% [45]. Chemotherapy, radiotherapy, and hormonal therapies can be considered to treat metastatic disease, but without clear evidence of their efficacy [46]. The management summary for PTs is shown in Figure 1.

Recurrence and metastasis

PTs recurrences are rarely in benign PTs, while common in borderline and malignant PTs, and are usually due to a positive resection margin status. Tumor size, stromal overgrowth, number of tumors, nuclear atypia, and pleomorphism, are considered risk factors for local recurrence [23,31]. Local recurrence varies between 0% and 60%, and in the majority of cases, within the first two years [21,31]. PTs distant metastases ranged from 25% to 40% in borderline and malignant types, but it is rare in benign type [35,47]. The commonest sites for metastasis are lungs, pleura, and bone [47].

Follow-up

Clinical assessment should be performed every six months, as most of the recurrence occurred within the first two years of treatment, and at the surgical site. The five-year survival rates are approximately 96% for benign type, 74% for borderline type, and 66% for malignant type [17,21].

## Conclusions

PTs are considered a rare type of breast tumor, which is difficult to diagnose preoperatively, and need the entire excised specimens to be diagnosed. Grading of PTs pathologically is important to predict the risk of recurrence, and survival rate. Benign and borderline PTs have a less aggressive disease course than malignant PT. Excision with negative margins is the recommended treatment. Adjuvant radiation therapy role in borderline and malignant PTs need to be investigated more.
